# A New Approach to Reduce Toxicities and to Improve Bioavailabilities of Platinum-Containing Anti-Cancer Nanodrugs

**DOI:** 10.1038/srep10881

**Published:** 2015-06-03

**Authors:** Li Liu, Qing Ye, Maggie Lu, Ya-Chin Lo, Yuan-Hung Hsu, Ming-Cheng Wei, Yu-Hsiang Chen, Shen-Chuan Lo, Shian-Jy Wang, Daniel J. Bain, Chien Ho

**Affiliations:** 1 Department of Biological Sciences, Carnegie Mellon University, Pittsburgh, PA; 2 Biomedical Technology and Device Research Laboratories, Industrial Technology Research Institute, Hsinchu, Taiwan; 3 Material and Chemical Research Laboratories, Industrial Technology Research Institute, Hsinchu, Taiwan; 4 Department of Geology and Planetary Science, University of Pittsburgh, Pittsburgh, PA.

## Abstract

Platinum (Pt) drugs are the most potent and commonly used anti-cancer chemotherapeutics. Nanoformulation of Pt drugs has the potential to improve the delivery to tumors and reduce toxic side effects. A major challenge for translating nanodrugs to clinical settings is their rapid clearance by the reticuloendothelial system (RES), hence increasing toxicities on off-target organs and reducing efficacy. We are reporting that an FDA approved parenteral nutrition source, Intralipid 20%, can help this problem. A dichloro (1, 2-diaminocyclohexane) platinum (II)-loaded and hyaluronic acid polymer-coated nanoparticle (DACHPt/HANP) is used in this study. A single dose of Intralipid (2 g/kg, clinical dosage) is administrated [intravenously (i. v.), clinical route] one hour before i.v. injection of DACHPt/HANP. This treatment can significantly reduce the toxicities of DACHPt/HANP in liver, spleen, and, interestingly, kidney. Intralipid can decrease Pt accumulation in the liver, spleen, and kidney by 20.4%, 42.5%, and 31.2% at 24-hr post nanodrug administration, respectively. The bioavailability of DACHPt/HANP increases by 18.7% and 9.4% during the first 5 and 24 hr, respectively.

Cancer remains the second most common cause of death in the US and 589,430 cancer deaths are projected to occur in 2015^1^. Platinum (Pt)-containing drugs (cisplatin, carboplatin, and oxaliplatin) are among the most widely used and most potent anti-cancer chemotherapeutic drugs for treatment of lung, colorectal, ovarian, breast, head and neck, bladder, and testicular cancers^2^,^3^,^4^. As is the case with other chemotherapeutic drugs, however, Pt drugs have their drawbacks, notably toxic side effects^2^,^3^,^4^. Side effects caused by off-target delivery to normal tissue and organs, notably nephrotoxcity in the kidneys, limit the use of Pt-based drugs^2^,^3^,^5^,^6^,^7^,^8^,^9^,^10^,^11^.

In order to significantly improve the therapeutic effects of current anti-cancer drugs, two problems need to be resolved urgently: (i) to improve delivery of the drug specifically to tumors and (ii) to reduce the toxic side effects of the drug. Nanomedicine, namely nanotechnology-based chemotherapeutics, has the potential to improve drug delivery and may generate new preventative, diagnostic, and therapeutic approaches to cancer in areas where improvements cannot be realized using existing technologies (http://nano.cancer.gov/). Nanocarriers tend to accumulate in solid tumors as a result of the enhanced permeability and retention (EPR) of macromolecules, thereby enhancing their anti-tumor or tumor-diagnosis activity^12^,^13^,^14^,^15^,^16^,^17^. The global anti-cancer nanomedicine market is predicted to grow from US$5.5 billion in 2011 to US$12.7 billion by 2016^12^. Several nanocarrier-based chemotherapeutics, such as Abraxane® and Doxil®, have been approved for treatment of several types of cancer^16^. Studies have shown that the therapeutic performance of oxaliplatin, which is a third generation Pt drug, can be improved by incorporating the central dichloro (1, 2-diaminocyclohexane) platinum (II) (DACHPt) motif into the core of these nanocarriers^18^,^19^,^20^,^21^.

A major limitation for both approved and in-development nanodrugs is their rapid clearance by the cells of the reticuloendothelial system (RES)/mononuclear phagocyte system, in particular liver and spleen, which can increase their toxicity to these off-target organs and reduce their efficacy^13^,^15^,^22^. Strategies that decrease RES uptake and increase the bioavailability of nanomedicines can improve tumor targeting and decrease the side effects. Many studies have been conducted to decrease RES clearance and to increase the tumor targeting of nanomedicines by modifying nanoparticle characteristics, such as the size, shape, charge, surface property, and composition^23^,^24^,^25^,^26^,^27^,^28^. Unfortunately, the total accumulation of the anti-cancer nanodrugs in the tumor represents a small fraction of total injected dose (1–10%). The majority (40–80%) of the injected nanomedicines end up in the liver and spleen^22^. Moreover, each new modification calls for thorough toxicity, pharmacology, and biomechanics studies before translating to a clinical setting.

Our strategy is to target the RES to temporarily blunt the uptake, i.e., to decrease the toxicities in liver and spleen and to increase the bioavailability of nanodrugs using Intralipid 20%, an FDA-approved fat emulsion used as parenteral nutrition source. The rational for this hypothesis is that the infusion of Intralipid has been reported to inhibit RES function by possibly inhibiting peritoneal clearance and impairing the phagocytic activity of Kupffer cells^29^. Kupffer cells in the liver play an important role in the uptake and metabolism of Intralipid^30^. Our recent findings also support this hypothesis. We have found that, in rodents, Intralipid can reduce RES uptake ~50% and increase blood half-life (t_1/2_) ~3-fold of nano- and micron-sized superparamagnetic iron-oxide particles^31^,^32^.

We have carried out this study with an improved Pt anti-cancer nanodrug, DACHPt-incorporated nanoparticles (NP), coated with hyaluronic acid polymer (HA) (DACHPt/HANP). We have found that a single, clinical dose of Intralipid (2 g/kg) can significantly reduce the toxic side effects of our Pt-containing nanodrug in liver, spleen, and kidney. Notably, our findings indicate that Intralipid pre-treatment decreases spleen enlargement, which has been reported as a serious side effect of Abraxane®. A single dose of Intralipid can decrease Pt accumulation in the liver (by 20.4%), spleen (42.5%), and kidney (39.3%) at 24-hr post nanodrug administration. Consequently, the bioavailability of the Pt-nanodrug increases by 18.7% during the first 5 hr and by 9.4% during 24 hr, respectively.

## Results

### Preparation and physical properties of DACHPt/HANP

DACHPt was encapsulated into DACHPt/HANP with a high efficiency of 85 ± 5%. The physical properties of DACHPt/HANP are summarized in Table 1. Detailed information on DACHPt/HANP characterization is shown in Figs. S1 and S2. DACHPt/HANP exhibits an average hydrodynamic diameter of 150 ± 30 nm (Fig. S1). The polydispersity index (PI) of DACHPt/HANP is 0.24 ± 0.05. The average Pt-core size is 19.1 ± 6.2 nm as measured by TEM (Fig. S2). At pH 6.5, DACHPt/HANP has a zeta potential (ζ) of −17.9 ± 5.5 mV.

### Intralipid Reduces Toxic Side Effects of Pt-Containing Nanodrug

Intralipid 20% was administered to Sprague Dawley (SD) rats at the clinical dose (2 g/kg) using the clinical route (i.e., intravenously) one hour before i.v. injection of DACHPt/HANP. At 24- and 72-hr post injection of DAHPt/HANP, blood samples were collected to determine serum alanine aminotransferase (ALT) activity and creatinine level to investigate liver and kidney damages. The tissue samples collected at 72-hr post injection were used for histological analysis. The tissue samples from naïve (SD) rats were used as controls.

#### Pathological Analysis and Terminal Deoxynucleotidyl Transferase dUTP Nick End Labeling (TUNEL) Assay for Apoptotic Cells in Liver

**Figure 1:**
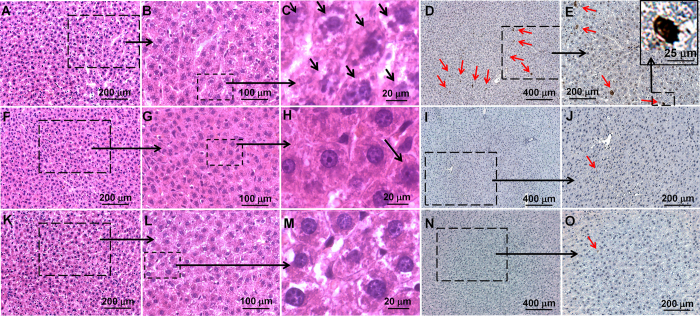
Intralipid reduces toxic side effects in liver caused by the anti-cancer nanodrug, DACHPt/HANP. Light microscopy images of H & E stained (**A**–**C**,**F**–**H**,**K**–**M**) and TUNEL stained liver tissue (**D**,**E**,**I**,**J**,**N**,**O**). (**A**–**E**) are from the liver tissues of DACHPt/HANP administrated, but no Intralipid treated, animals; (**F**–**J**) are from the liver tissues of Intralipid pre-treated animals; (**K**–**O**) are from the liver tissues of naïve animals. (**C**) is an example of enlarged view of (**B**) which is enlarged from part of (**A**). So is for (**H**,**M**,**E**,**J**,**O**). Black arrows on (**C**,**H**) indicate cell necrosis; red arrows on (**D**,**E**) indicate cell apoptosis.

Light microscopic images of hematoxylin/eosin (H & E) stained liver tissue sections are shown in Fig. 1A–C,F–H,K–M. Images of TUNEL stained liver tissue sections are shown in Fig. 1D,E,I,J,N,O. With DACHPt/HANP administration, but no Intralipid pre-treatment, the pathological changes in the liver tissue are characterized by necrosis, as indicated by black arrows in Fig. 1C, which is an example of enlarged view from Fig. 1A,B. Apoptotic cells are observed with TUNEL staining, as indicated by red arrows in Fig. 1D,E, from the liver tissue of this treatment group. An enlarged view of an apoptotic cell is shown as an example in Fig. 1E. These damages are significantly reduced upon Intralipid pre-treatment. The liver tissue sections from the Intralipid pre-treated group are shown in Fig. 1F–J. Very few cell necroses (black arrow in Fig. 1H) and apoptotic cells (red arrows in Fig. 1J) are observed, comparable to the liver tissues of naïve rats (Fig. 1K–O).

#### Spleen Enlargement

**Figure 2:**
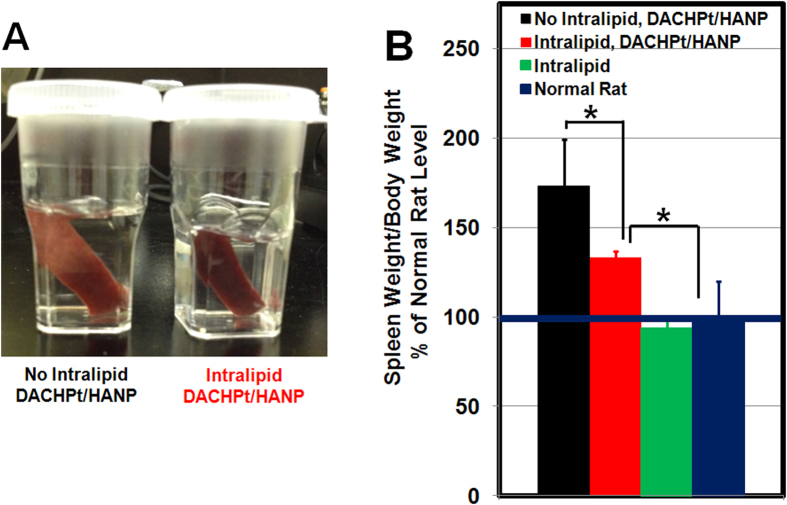
Intralipid pre-treatment can reduce spleen swelling significantly: (A) picture of the spleens from DACHPt/HANP treated, without or with Intralipid treated, SD rats; and (B) the changes in spleen weight/body weight ratio upon Intralipid treatment.The ratio from a naïve SD rat is treated as 100%. ^*^p < 0.05.

Spleen swelling and enlargement are observed from DACHPt/HANP-treated animals, when the animals are sacrificed 72-hr post nanodrug administration (Fig. 2A **left**). Intralipid pre-treatment appears to reduce spleen swelling (Fig. 2A **right**). The ratio of spleen weight/body weight for a naïve Sprague Dawley (SD) rat is 0.31 ± 0.06 (n = 3). Intralipid treatment does not cause spleen swelling with the ratio of 0.28 ± 0.02 (n = 3). The ratio from a DACHPt/HANP treated SD rat is 0.53 ± 0.08 (n = 3). Upon Intralipid pre-treatment, this ratio reduces to 0.4 ± 0.008 (n = 3). In Fig. 2B, the ratios are shown as the percentage of the normal level.

#### Pathological and TUNEL Assay Analyses of Spleen

**Figure 3:**
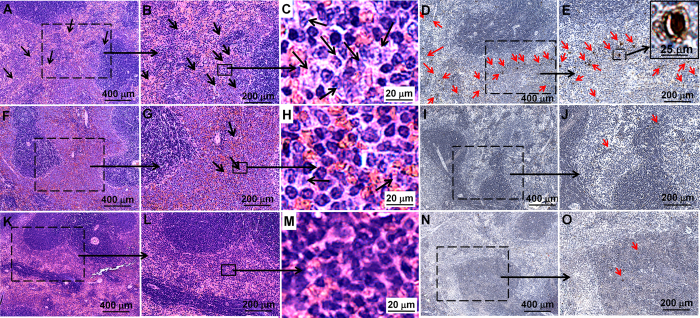
Intralipid reduces toxic side effects in spleen caused by DACHPt/HANP. Light microscopy images of H & E stained (**A**–**C**,**F**–**H**,**K**–**M**) and TUNEL stained spleen tissue (**D**,**E**,**I**–**J**,**N**–**O**). (**A**–**E**) are from the spleen tissues of DACHPt/HANP administrated, but no Intralipid treated, animals; (**F**–**J**) are from Intralipid pre-treated animals; (**K**–**O**) are from naïve healthy animals. Black arrows on (**A**) indicate concurrent abnormal proliferation of mononuclear cells; black arrows on (**B**,**C**,**G**,**H**) indicate cell necrosis; red arrows on (**D**,**E**) indicate cell apoptosis.

Light microscopic images of H & E stained (Fig. 3A–C,F–H,K–M) and TUNEL stained (Fig. 3D,E,I,J,N,O) spleen tissue sections are shown in Fig. 3. With DACHPt/HANP administration, but no Intralipid pre-treatment, the pathological changes in the spleen tissue are characterized by concurrent abnormal proliferation of mononuclear cells as indicated by black arrows on Fig. 3A and necrosis as indicated by black arrows on Fig. 3B,C. Morphological changes and enlarged size are also observed. TUNEL staining of spleen tissue from DACHPt/HANP treatment reveals a large amount of apoptotic cells (Fig. 3D,E). In contrast, uniformly distributed mononuclear cells (Fig. 3F), few necrotic (Fig. 3G,H), and few apoptotic (Fig. 3I,J) spleen cells are detected from the Intralipid pre-treatment group, which is similar to that of naïve rats (Fig. 3K–O).

#### Pathological and TUNEL Assay Analyses of Kidney

**Figure 4:**
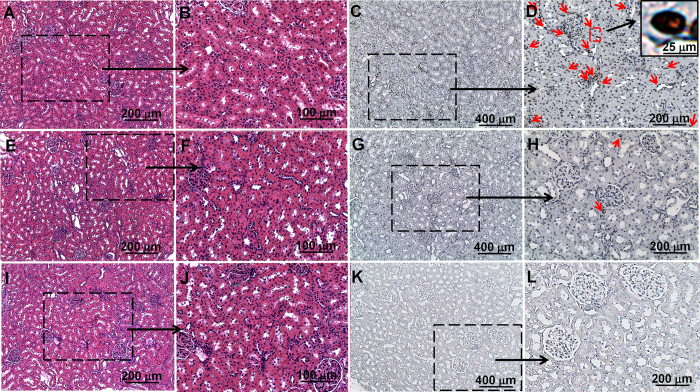
Intralipid reduces toxic side effects in kidney caused by DACHPt/HANP. Light microscopy images of H & E stained (**A**,**B**,**E**–**F**,**I**,**J**) and TUNEL stained spleen tissue (**C**,**D**, **G**,**H**,**K**,**L**). (**A**–**D**) are from the kidney tissues of DACHPt/HANP administrated, but no Intralipid treated, animals; (**E**–**H**) are Intralipid pre-treated animals; (**I**–**L**) are from naïve healthy animals. Red arrows on (**D**,**H**) indicate cell apoptosis.

Intralipid also protects kidney cells from the damage caused by the Pt-nanodrug. With Intralipid pre-treatment, the apoptotic cells in kidney, caused by the treatment of DACHPt/HANP, decreased dramatically (Fig. 4D vs H, red arrows). Light microscopic images of H & E stained kidney tissue, with or without Intralipid pre-treatment, look similar (Fig. 4B,F).

#### ALT Activity and Creatinine Colorimetric Assays to Assess Liver and Kidney Damages

**Figure 5:**
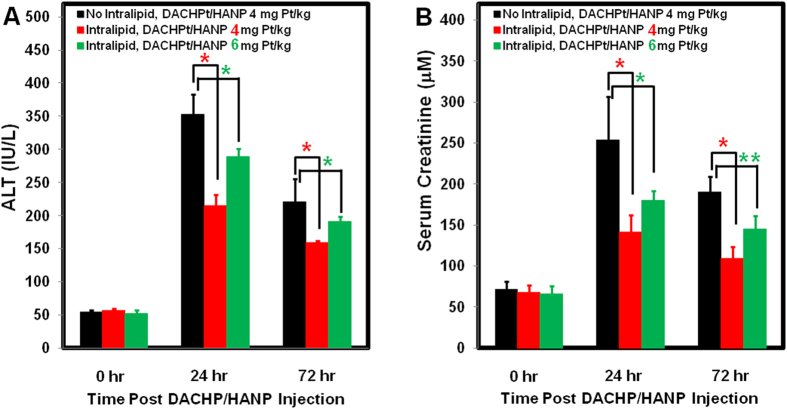
Effects of Intralipid pre-treatment on the serum ALT activities (A) and creatinine levels (B) in DACHPt/HANP treated rats. When the rats are treated with 4 mg Pt/kg of the nanodrug, Intralipid pre-treatment group shows significantly lower serum ALT activity and creatinine level (**A**,**B**). The group, which is pre-treated with Intralipid followed by the treatment of a higher dosage (6 mg Pt/kg) of DACHPt/HANP, exhibits lower ALT activity (**A**) and creatinine level (**B**) than the group, which is treated with 4 mg Pt/kg of the nanodrug, but no Intralipid. ^*^p < 0.001; ^**^p < 0.05.

The serum ALT activity is 54.4 ± 3.3 IU/L for naïve rats. Intralipid treatment does not alter ALT activity (57.1 ± 2.2 IU/L) (Fig. 5A). With no Intralipid protection, the serum ALT activities elevate to 353.2 ± 29.9 IU/L and 220.4 ± 34.9 IU/L at 24- and 72-hr post Pt-nanodrug injection, respectively. With Intralipid pre-treatment, serum ALT activities are 214.9 ± 16.5 IU/L and 159.5 ± 3.1 IU/L at 24 hr and 72 hr, indicating that Intralipid reduces the hepatocellular damages from the Pt-nanodrug. This result is consistent with our findings in the liver histological studies as shown in Fig. 1.

Consistent with our pathological findings in kidney (Fig. 4), Intralipid pre-treatment also decreases serum creatinine level significantly (Fig. 5B). At 24- and 72-hr post DACHPt/HANP administration, the creatinine levels increase to 253.6 ± 53.1 μM and 190.2 ± 19.2 μM, respectively. With Intralipid pre-treatment, the creatinine levels are 141.0 ± 21.1 μM and 109.0 ± 14.8 μM, respectively, indicating a reduction of the kidney damage.

In order to show the potency of this Intralipid protective effects, the rats (n = 3) were pre-treated with Intralipid followed by a higher dosage, 6 mg Pt/kg, of DACHPt/HANP. At 24- and 72-hr post nanodrug treatment, serum ALT activities are 289.2 ± 11.3 IU/L and 191.5 ± 6.9 IU/L, respectively (Fig. 5A); creatinine levels are 180.2 ± 11.3 μM and 145.2 ± 16.2±μM, respectively (Fig. 5B). These activities and levels are all significantly lower than the group treated with lower dosage of the nanodrug (4 mg Pt/kg), but no Intralipid pre-treatment.

### Changes of DACHPt/HANP Accumulation in Tissues upon Intralipid Pre-Treatment

The Pt concentration in tissue (spleen, liver, and kidney) and blood of naïve animals or Intralipid along or phosphate-buffered-saline (PBS) treated animals is below 0.01 part per million (ppm).

#### DACHPt/HANP Accumulation in Liver

**Figure 6:**
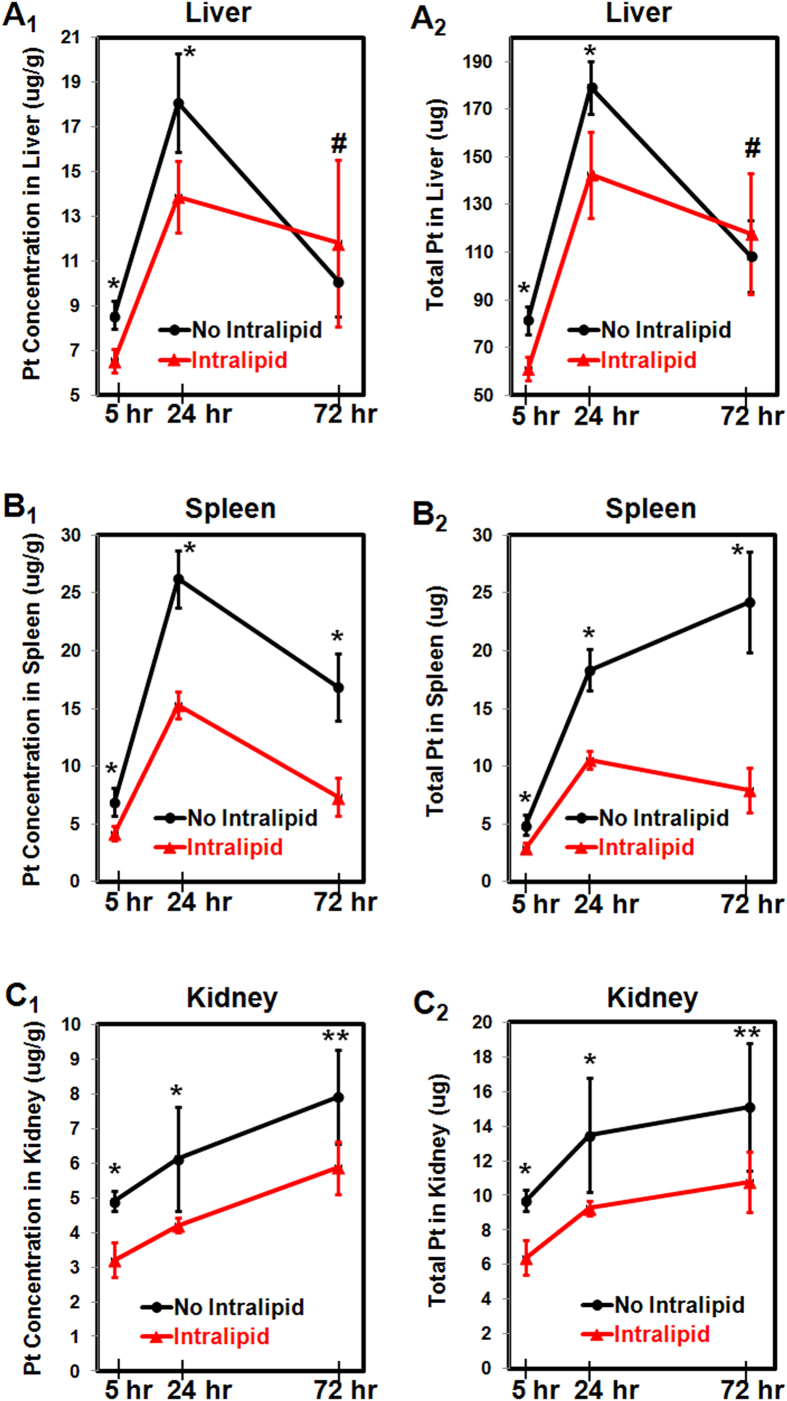
Changes in concentrations (A_1_,B_1_,C_1_) and total amounts (A_2_,B_2_,C_2_) of Pt in liver (A_1_,A_2_) spleen (B_1_,B_2_) and kidney (C_1_,C_2_) upon Intralipid pre-treatment, at 5-, 24-, and 72-hr post DACHPt/HANP administration. *P* values represent the significance differences from the concentration or amount of Pt in the tissue from the Intralipid pre-treated group at the same time point. ^*^p < 0.001; ^**^p < 0.01; ^#^*p* > 0.1.

With DACHPt/HANP administration, the Pt concentrations in liver are 8.6 ± 0.6 and 18.1 ± 2.2 (μg/g wet weight) at 5- and 24-hr post injection (Fig. 6A1). These translate into 81.6 ± 5.9 and 179.0 ± 11.2 μg Pt in the liver (Fig. 6A2). With Intralipid pre-treatment, the Pt concentrations in the liver decrease to 6.6 ± 0.5 and 13.9 ± 1.6 (μg/g wet weight) at 5- and 24-hr post DACHPt/HANP injection (Fig. 6A1), respectively. The total amounts of Pt decrease to 61.2 ± 4.2 and 142.5 ± 18 μg (Fig. 6A2), respectively. Thus, one single administration of Intralipid can significantly decrease liver accumulation of the nanodrug by 24.9% and 20.4% at 5- and 24-hr post injection, respectively.

With the drug being metabolized in the liver, the Pt concentrations reach similar level at 72 hr: 10.1 ± 1.6 and 11.8 ± 3.7 (μg/g wet weight), without- and with-Intralipid pre-treatment, respectively.

#### DACHPt/HANP Accumulation in Spleen

Figure 6B1 show the changes in the spleen accumulation of the DACHPt/HANP upon Intralipid pre-treatment. With DACHPt/HANP administration, the Pt concentrations in spleen are 6.9 ± 1.2, 26.2 ± 2.5, and 16.9 ± 2.9 (μg/g wet weight) at 5-, 24-, and 72-hr post injection, respectively (Fig. 6B1). These translate into 4.9 ± 0.9, 18.3 ± 1.8, 24.2 ± 4.4 μg Pt in spleen, respectively (Fig. 6B2). With Intralipid pre-treatment, the Pt concentration in the spleen decreases to 4.2 ± 0.6, 15.3 ± 1.2, and 7.3 ± 1.6 (μg/g wet weight), respectively (Fig. 6B1) and the total amount of Pt in the spleen decreases to 2.9 ± 0.4, 10.6 ± 0.8, and 7.9 ± 1.9 μg, respectively (Fig. 6B2) at these three time points. Thus, one single administration of Intralipid can significantly decrease spleen uptake of the nanodrug by 40.1%, 42.4, and 67.2% at 5-, 24-, and 72-hr post administration.

#### DACHPt/HANP Accumulation in Kidney

We have observed that the Pt accumulations in kidney also decrease upon Intralipid pre-treatment (Fig. 6C1). With no Intralipid pre-treatment, the Pt concentration in kidney is 4.9 ± 0.3, 6.1 ± 1.5, and 7.9 ± 1.4 (μg/g wet weight) at 5-, 24- and 72-hr post DACHPt/HANP injection (Fig. 6C1). These translate into 9.7 ± 0.6, 13.5 ± 3.8, and 15.1 ± 3.7 μg Pt in kidney (Fig. 6C2). With Intralipid pre-treatment, the Pt concentrations in kidney decrease to 3.2 ± 0.5, 4.2 ± 0.5, and 5.9 ± 0.7 (μg/g wet weight) (Fig. 6C1) and total amounts of Pt in kidney decrease to 6.4 ± 1.0, 9.3 ± 0.4, and 10.8 ± 1.7 μg (Fig. 6C2) at 5-, 24- and 72-hr post DACHPt/HANP injection. Thus, Intralipid pre-treatment can significantly decrease the Pt drug accumulation in the kidney by 34.0, 31.2, and 28.7% at 5-, 24-, and 72-hr post DACHPt/HANP administration, respectively.

### Blood Clearance and Bioavailability

**Figure 7:**
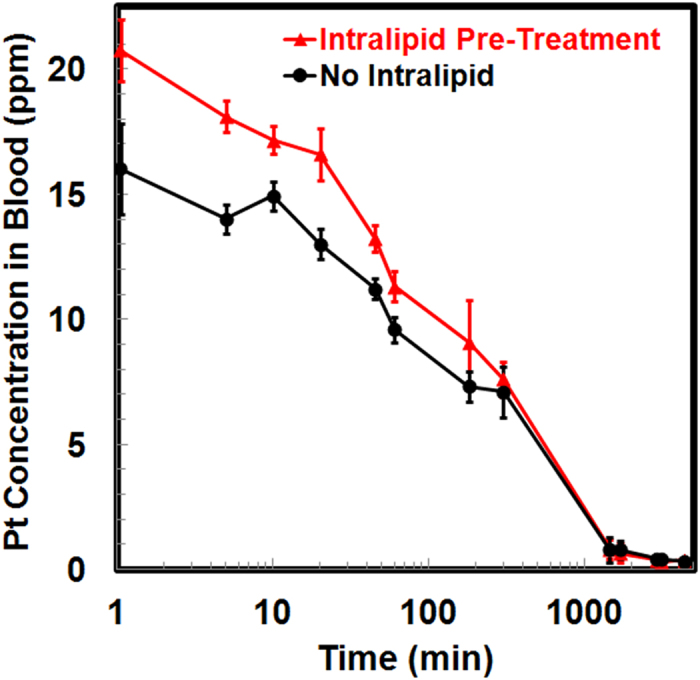
Changes in the Pt concentrations in blood upon Intralipid pre-treatment during 72 hr. X-axis represents the duration post DACHPt/HANP injection, in logarithmic scale (base: 10).

Changes in the Pt concentrations in blood upon Intralipid pre-treatment are shown in Fig. 7. The bioavailability of the Pt-drug is calculated by the area under the curve (AUC), namely the integral of the Pt concentration-time curve, using the trapezoidal rule. A single administration of Intralipid can increase the bioavailability of the Pt drug by 18.7% during the first 5 hr (*p* < 0.0001) and by 9.4% during 24 hr (*p* < 0.001) (Fig. S3). This finding indicates that Intralipid can change the clearance and increase the bioavailability of the nanodrug.

## Discussion

After several decades, the research seeking for less toxic Pt drugs and better drug delivery methods, which can decrease the associated side effects and improve the anti-cancer efficacy as well as the quality of life of the patients, still goes on. We have found a new approach to reduce the side effects and increase the bioavailabilities of an anti-cancer Pt-containing nanodrug (DACHPt/HANP), by using an “old” FDA approved agent, Intralipid. Since the approval of cisplatin in 1979, Pt-based drugs, including carboplatin and oxaliplatin (second and third generation), have become the most potent as well as the most widely prescribed anti-cancer drugs^2^. Unfortunately, its continuous use is greatly limited by dose limiting toxicities, partial anti-tumor response in most patients, development of drug resistance, and tumor relapse^2^,^3^,^5^,^8^,^9^,^10^,^33^. Nanocarrier-based drug delivery may generate new therapeutic approaches for Pt-drugs. Pt-based nanodrugs are providing encouraging preclinical and clinical results and may facilitate the development of the next generation of Pt chemotherapy^18^,^19^,^34^. However, the important questions of how to decrease the RES uptake, which accounts for 40–80% of injected nanodrugs, and how to reduce the toxic side effects caused by this off-target uptake, still need answers. Our studies show that Intralipid pre-treatment can be used to reduce RES uptake and side effects, and improve the bioavailability and clinical applications of Pt-containing nanodrugs. Moreover, we have observed that Intralipid treatment can decrease Pt accumulation in kidney, thus reducing nephrotoxicity of the Pt drug.

Current approved anti-cancer nanodrugs, namely Abraxane®, Doxil®, DaunoXome®, and DepoCyt®, work by loading traditional cancer chemotherapeutics into nanocarriers. These chemotherapeutics are believed to inhibit mitosis (paclitaxel loaded in Abraxane®), cause DNA intercalation (doxorubicin and daunorubicin loaded in Doxil® and DaunoXome®), and interfere with DNA synthesis (cytarabine loaded in DepoCyt®)^12^,^16^. Thus, the accumulation of these drugs in mononuclear phagocytic cells in the liver and spleen would cause toxic side effects. For many nanomedicines, the toxicity in the mononuclear phagocyte system is the killer for further development^35^. DACHPt loaded in HANP induces cancer cell apoptosis by causing cross-linking of DNA and DNA-protein. DACHPt-loaded polymeric micelles have been reported to cause liver toxicity^20^. When animals were sacrificed at 72-hr post DACHPt/HANP administration, we observed dramatic swelling and enlargement of the spleen from DACHPt/HANP-treated animals. Pre-treatment with Intralipid 20% (clinical dose, 2 g/kg) can reduce spleen swelling significantly (Fig. 2). Pathological and cell apoptosis analyses reveal that Intralipid can be used to decrease the toxic side effects of our anti-cancer nanodrug in the mononuclear phagocyte system (Figs. 1 and 3). The serum ALT assay also indicates that Intralipid can protect liver from the damage caused by the nanodrug off-target accumulation (Fig. 5A).

In a previous study^31^, we have found that in rodents, Intralipid can reduce RES uptake by ~50% of nano- and micron-sized particles in which MRI contrast agents are loaded. The RES plays an important role in the uptake and metabolism of Intralipid^30^,^36^. The blood half-life of Intralipid 20% administered by intravenous bolus in rats is 8.7 ± 3.0 min^30^,^36^. The diameter of the Intralipid particles range from 200 to 1000 nm^37^. As shown in Fig. 6A,B, Intralipid pre-treatment decreases the liver and spleen uptake of the nanodrug by 20.4% and 42.5% at 24-hr post nanodrug administration, respectively.

Interestingly, Intralipid pre-treatment can also decrease the Pt accumulation in the kidney (Fig. 6C1). Nephrotoxicity is one of the most severe side effects of current Pt drugs^2^,^3^,^6^,^8^,^9^,^10^. DACHPt/HANP nanodrug is designed to increase the concentration and prolong the half-life of DACHPt at tumor sites and to decrease the side effects like nephrotoxicity. Although our Intralipid therapy was originally designed to decrease the RES uptake of the nanodrug, Intralipid pre-treatment could also decrease the Pt drug (DACHPt and/or DACHPt/HANP) accumulation in kidney by 28.7% at 72 hr. Regarding the Pt concentration, we should keep in mind that two components contribute to the Pt concentration: the DAHPt/HANP nanodrug and the DAHPt molecule, which is released from the polymer coating. As a consequence, Intralipid also decreases the nephrotoxicity of the Pt-nanodrug (Figs. 4 and 5B).

This protective effect of Intralipid is so potent that the rats from a higher dosage treatment (6 mg Pt/kg of DACHPt/HANP) exhibit a less hepatocellular and nephrocellular damages (Fig. 5A,B). This indicates that, using Intralipid, the clinicians might be able to give the patients more anti-cancer nanodrugs to kill the tumors with less toxic side effects!

Intralipid can change the clearance and increase the bioavailability of the nanodrug. Our results show that a single dose of Intralipid can increase the bioavailability of DACHPt/HANP by 18.7% during the first 5 hr (Fig. 7). It has been reported that after i.v. administration of Intralipid, the circulating ketone bodies increased ~100% in 30 min, which indicates an active metabolism of Intralipid by the liver^30^. This active metabolism might explain the decrease of the effectiveness of Intralipid after 5 hr. To increase and prolong the effectiveness of Intralipid, the administration routes, dosages, and time courses of Intralipid treatment need to be optimized in a future study. Multiple administrations of Intralipid may be necessary.

Moreover, the development of targeted nanomedicine has made an important impact in new drug development in neurology^38^, cardiology^39^, and inflammation^33^. The EPR effect is found not only in cancer, but also in a wide range of inflammatory diseases, such as atherosclerosis^40^,^41^,^42^. Thus, our findings for Intralipid pre-treatment could have broad applications besides cancer.

## Concluding Remarks

Our study shows that Intralipid can be used to reduce the toxic side effects of Pt-containing anti-cancer nanodrugs in the liver, spleen, and kidney, and also to improve the bioavailability of the nanodrug. Our approach is also a general one applicable to any approved and in-development nanodrugs to improve their bioavailability and to decrease their toxic side effects, without any new modification of the nanodrugs and/or the nanocarriers. Intralipid has been used for over 40 years as a safe source of parenteral nutrition for patients and so can readily translate to clinical use. The outcome of this study has the potential to decrease the toxic side effects of anti-cancer nanodrugs and other nanodrugs, and therefore to reduce human suffering. Also, increasing efficacy could lead to a reduction of the dosage of these expensive drugs: the average cost per dose is US$4,000–6,000. Thus, our findings for the use of Intralipid with nanodrugs can also lead to the reduction of healthcare costs as well as to the improvement of the quality of life for patients who undergo the therapeutic treatment.

## Materials and Methods

### Materials and Animals

Intralipid 20% was purchased from Fresenius Kabi (Bad Homburg, Germany). Dichloro(1,2-diamminocyclohexane) platinum(II) (DACHPtCl_2_), AgNO_3_, and the platinum (Pt) standard were purchased from Sigma-Aldrich (St. Louis, MO). Phosphate-buffered-saline (PBS) was obtained from Mediatech (Manassas, VA).

Male SD rats with an indwelling jugular vein catheter implanted were purchased from Harlan Laboratories (Indianapolis, IN). All experiments involving animal subjects were approved by the Institutional Animal Care and Use Committee of Carnegie Mellon University. Animal care was provided in accordance with the Guide for the Care and Use of Laboratory Animals.

### Preparation and Physical Properties of DACHPt/HANP

DACHPt/HANP was prepared with modified procedures from a previously described method^18^. In brief, DACHPtCl_2_ was mixed with silver nitrate ([AgNO_3_]/[DACHPt] = 2) to form an aqueous complex. The solution was kept in the dark at 25 °C for 24 hr. AgCl precipitates were removed by centrifugation followed by filtration through a 0.22-μm hydrophilic polyvinylidene fluoride (PVDF) membrane (Millipore, Billerica, MA). Subsequently, HA/Boc-His/PEG graft copolymers, comprising hyaluronic acid (Mw = 16 kD), were added to the aqueous complex of DACHPt at a 0.33 molar ratio of DACHPt to carboxylate groups of the HA modified polymers. The mixture was stirred in the dark for three days at 25 °C. The reaction mixture was sonicated and then purified by ultrafiltration against deionized water to remove uncoordinated DACHPt. The product was filtered through a 0.22-μm PVDF membrane and lyophilized with 10% trehalose.

The particle size and PI of DACHPt/HANP was determined by dynamic light scattering using a ZetaPlus (Brookhaven, Holtsville, NY). Zeta potential was measured by the laser Doppler anemometry (Zeta Plus zeta potential analyzer, Brookhaven Instruments Corporation).

TEM images were taken by using a Cryo transmission electron microscope (Cryo-TEM) [JEM-2100 (JEOL, Tokyo, Japan)] operated at 200 kV with attachment of energy dispersive spectroscopy (EDS). A droplet of DACHPt/HANP solution was adsorbed on a cleaned carbon film supported copper grid. After excess sample was removed, phosphotungstic acid (Merck) was used as negative stain reagent to improve the image contrast. TEM grid was dried in the contamination-free environment and reserved in the electronic dry cabinet for further TEM analysis.

### Encapsulation Efficiency of DACHPt in DACHPt/HANP

In order to determine the encapsulation efficiency of DACHPt in the nanocomplex, the amount of Pt were quantified by inductively coupled plasma-optical emission spectrometry (ICP-OES) in preparation processes. Encapsulation efficiency (EE %) was calculated using below formula:
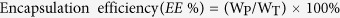
where W_P_ is the total amount of Pt after purification by passing through a 0.22 μm filter and W_T_ is the total quantity of Pt determined before purification.

### Experimental Design

Male SD rats, with body weights between 250 and 280 g, were used. Intralipid 20% was administered by intravenous injection at a clinical dose of 2 g/kg. PBS was administered to control animals. After one hr, DACHPt/HANP (2 mg Pt/kg for bioavailability and biodistribution studies, n = 14 for Intralipid pre-treatment group and n = 14 for control group; 4 mg Pt/kg for toxicity studies, n = 3 for Intralipid pre-treatment group and n = 3 for control group; 6 mg Pt/kg for another toxicity study to test the serum ALT activity and creatinine level, n = 3 for Intralipid pre-treatment group) was injected intravenously. Blood samples were collected at different time points to determine the bioavailability of DACHPt/HANP. At 5-, 24-, and 72-hr post injection of DACHPt/HANP, tissues (liver, spleen, and kidney) were collected for the Pt-level determination. The tissue samples collected at 72-hr post injection were used for histological analysis.

### Blood Bioavailability

An indwelling jugular vein catheter was used for repeated blood samplings. Blood samples (100 μL) were collected at different time points to determine the changes of bioavailability of DACHPt/HANP upon Intralipid treatment. Blood was sampled after DACHPt/HANP injection at 1, 5, 10, 20, 45, and 60 min, 3, 5, 24, 28, 48, 52, and 72 hr. The blood samples were decomposed in HNO_3_ (0.5 mL) at 60 °C overnight and re-dissolved in 0.5 mL of 2 N HCl^18^,^20^,^43^. Suitable dilutions were prepared using 5% HCl to reach a final Pt concentration in the range of 0.02 to 1 part per million (ppm). Samples were analyzed for Pt concentration by inductively coupled plasma-mass spectrometry (ICP-MS) [NexION 300X (PerkinElmer, Waltham, MA)], with modified procedures from our previous studies^31^,^32^. ^194^Pt, ^195^Pt, and ^196^Pt isotopes were analyzed and similar results were obtained from the measurement of these three isotopes. The Pt concentrations shown in this manuscript were calculated from the measurements of ^194^Pt. Bioavailability was calculated by the area under the curve (AUC), namely the integral of the concentration-time curve, using the trapezoidal rule with the use of KaleidaGraph 4.1 (Synergy Software, Reading, PA).

### Pt Levels in Tissues

The wet weight of each tissue sample was recorded. Tissue homogenate (0.5 mL) was decomposed in HNO_3_ (1 mL) at 60 °C overnight. The rest of the tissue was fixed in 4% paraformaldehyde for histological analyses. The HNO_3_-digested samples were evaporated and then re-dissolved in 0.5 mL of 2 N HCl^43^. The Pt concentrations in the solution were analyzed by ICP-MS as described above.

### Pathological Analysis and TUNEL Assay

Histological examinations and TUNEL assays were performed by the Transplantation Pathology Laboratory of the University of Pittsburgh Medical Center (Pittsburgh, PA). Paraffin-embedded 5-μm sections were stained with hematoxylin/eosin (H & E), or performed TUNEL staining. For histopathological diagnosis, slides were examined by light microscopy and photomicrographs were taken using a Moticam 2300 camera mounted on an Olympus Provis microscope with Mtic Images Plus 2.0 software.

### ALT Activity Assay and Creatinine Colorimetric Assay

The activity of ALT in serum was measured by using the ALT Activity Assay Kit purchased from Sigma-Aldrich, according to the supplier’s protocol. Serum creatinine level was measured by using the Creatinine Colorimetric/Fluorometric Assay Kit purchased from BioVision.

### Statistical Analysis

Statistical analysis was carried out with Student’s *t* test. A *p* value < 0.05 was considered statistically significant.

## Additional Information

**How to cite this article**: Liu, L. *et al.* A New Approach to Reduce Toxicities and to Improve Bioavailabilities of Platinum-Containing Anti-Cancer Nanodrugs. *Sci. Rep.* **5**, 10881; doi: 10.1038/srep10881 (2015).

## Supplementary Material

Supplementary Information

## Figures and Tables

**Table 1:** Physical properties of DACHPt/HANP.

	**Hydrodynamic Diameter (nm)**	**PI**	**Core Diameter (nm)**	**Zeta Potential pH 6.5 (mV)**
DACHPt/HANP	150 ± 30	0.24 ± 0.05	19.1 ± 6.2	−17.9 ± 5.5
