# Supplementary material for: A New Approach to Reduce Toxicities and to Improve Bioavailabilities of Platinum-Containing Anti-Cancer Nanodrugs
Source: Sci Rep. 2015 Jun 3;5:10881. doi: 10.1038/srep10881 (PMC4454134; doi:10.1038/srep10881)
Supplement: Supplementary Information [file srep10881-s1.pdf]

Supplementary Information

A New Approach to Reduce Toxicities and to Improve Bioavailabilities of Platinum-Containing Anti-Cancer Nanodrugs

Li Liu [1†], Qing Ye [1†], Maggie Lu [2], Ya-Chin Lo [2], Yuan-Hung Hsu [2], Ming-Cheng Wei [2], Yu-Hsiang Chen[3], Shen-Chuan Lo[3], Shian-Jy Wang [3], Daniel J. Bain [4], Chien Ho [1*]

[1] Department of Biological Sciences, Carnegie Mellon University, Pittsburgh, PA; [2] Biomedical Technology and Device Research Laboratories, Industrial Technology Research Institute, Hsinchu, Taiwan; [3] Material and Chemical Research Laboratories, Industrial Technology Research Institute, Hsinchu, Taiwan; [4] Department of Geology and Planetary Science, University of Pittsburgh, Pittsburgh, PA.

[†]LL and QY contributed equally.

[*] Correspondence author at: Chien Ho, Department of Biological Sciences, Carnegie Mellon University, 4400 Fifth Ave, Pittsburgh, PA 15213, USA; Tel: +1 412 268 3395; Fax: +1 412 268 7083; E-mail address: chienho@andrew.cmu.edu

Figures

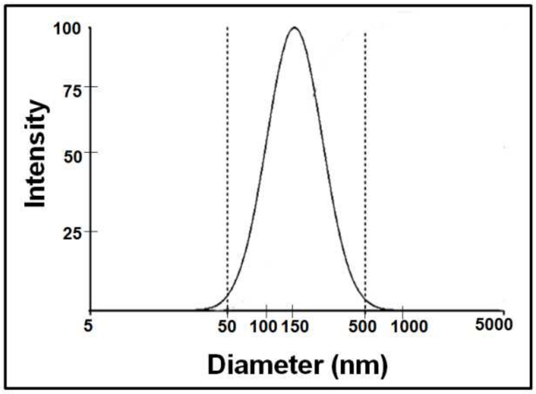

Fig S1. Dynamic light scattering (DLS) analysis of the hydrodynamic diameter of DACHPt/HANP.

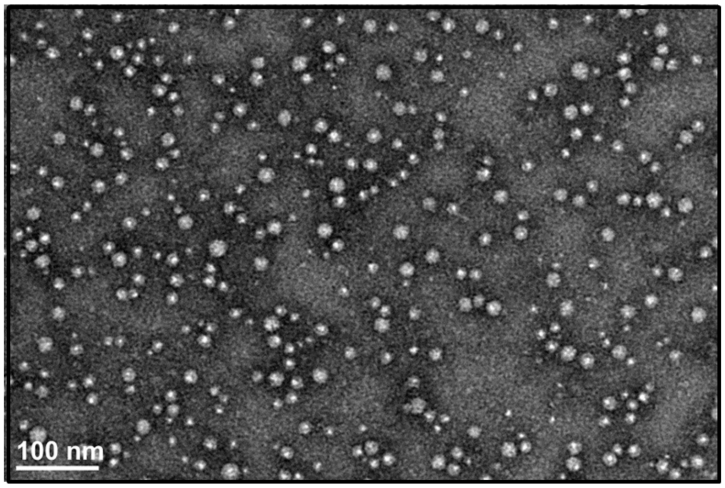

Fig S2. Cryo transmission electron microscope (Cryo-TEM) image of DACHPt/HANP.

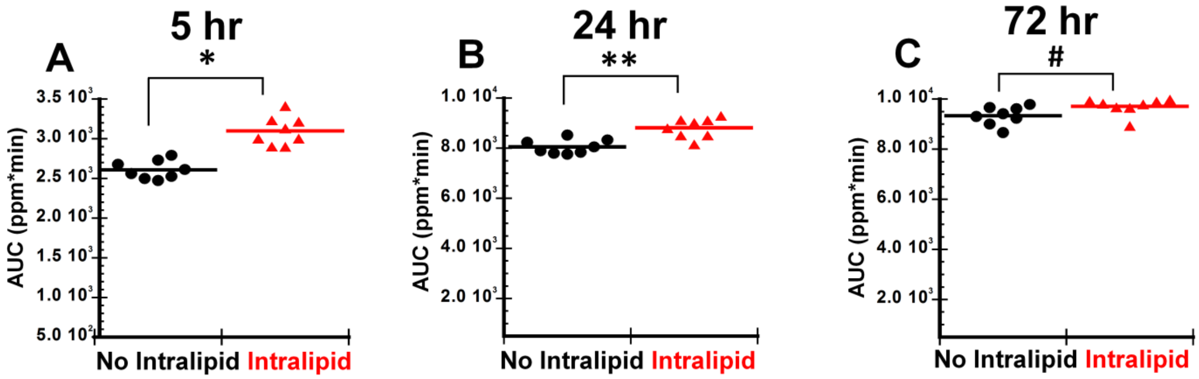

Fig S3. Changes in the bioavailability of DACHPt/HANP nanodrug [calculated by the area under the curve (AUC), by the trapezoidal rule, using KaleidaGraph 4.1 (Synergy Software, Reading, PA)] upon Intralipid pre-treatment: (A) 5 hr; (B) 24 hr; (C) 72 hr after the nanodrug administration. $^{*}p < 0.0001$; $^{**}p < 0.001$; $^{\#}p > 0.05$.
